# Advances in targeting tumor microenvironment for immunotherapy

**DOI:** 10.3389/fimmu.2024.1472772

**Published:** 2024-10-03

**Authors:** Lugang Wang, Liubo Zhang, Zhen Zhang, Peng Wu, Yi Zhang, Xinfeng Chen

**Affiliations:** ^1^ Biotherapy Center, The First Affiliated Hospital of Zhengzhou University, Zhengzhou, Henan, China; ^2^ Department of Oncology, The First Affiliated Hospital of Zhengzhou University, Zhengzhou, Henan, China; ^3^ State Key Laboratory of Esophageal Cancer Prevention & Treatment, Zhengzhou, Henan, China; ^4^ School of Life Sciences, Zhengzhou University, Zhengzhou, Henan, China; ^5^ Engineering Key Laboratory for Cell Therapy of Henan Province, Zhengzhou, Henan, China; ^6^ Tianjian Laboratory of Advanced Biomedical Sciences, Academy of Medical Sciences, Zhengzhou University, Zhengzhou, Henan, China

**Keywords:** tumor microenvironment, immunosuppression, targeted, immunotherapy, cancer

## Abstract

The tumor microenvironment (TME) provides essential conditions for the occurrence, invasion, and spread of cancer cells. Initial research has uncovered immunosuppressive properties of the TME, which include low oxygen levels (hypoxia), acidic conditions (low pH), increased interstitial pressure, heightened permeability of tumor vasculature, and an inflammatory microenvironment. The presence of various immunosuppressive components leads to immune evasion and affects immunotherapy efficacy. This indicates the potential value of targeting the TME in cancer immunotherapy. Therefore, TME remodeling has become an effective method for enhancing host immune responses against tumors. In this study, we elaborate on the characteristics and composition of the TME and how it weakens immune surveillance and summarize targeted therapeutic strategies for regulating the TME.

## 1 Introduction

In the history of the struggle against cancer, from the initial belief that cancer is a tumor to its recognition as a complex systemic ailment, the use of the body’s immune system to specifically target and kill tumors has evolved (1). The TME, the latest research direction in cancer studies, has been confirmed by multiple studies as a key player in the development of tumors. The TME symbolizes the environment of cancer cells and embodies an intricate biome comprising vascular networks, extracellular matrices, immune cells, and various adjacent elements enveloping cancerous cells. Elements within the TME communicate and mutually regulate both cellular and noncellular components (2, 3). The “dysfunctional” state of tumor blood vessels fosters features such as a lack of oxygen and acidic conditions within the TME, creating an immunosuppressive microenvironment. Furthermore, the extracellular matrix, along with fibroblast-produced fibronectin and proteoglycans, provides crucial support for the proliferation and mobility of cancerous cells, influencing the invasive behavior and metastatic capabilities of tumors.

Recent years have seen notable advancements in cancer immunotherapy, specifically through the utilization of immune checkpoint inhibitors (ICIs) targeting programmed cell death protein 1 (PD-1) and its ligand (PD-L1), offering hope to many patients with advanced cancer (1). Enhancing the effectiveness of immunotherapy remains a challenge, given that the immunosuppressive features of TMEs enable tumors to evade immune detection, thereby rendering current treatment strategies ineffective (4). Hence, a thorough study of the composition and attributes of the TME, along with the targeted exploration of therapeutic approaches, is of great significance. This study focuses on exploring the intricate mechanisms by which the TME influences cancer progression, examining insights from foundational studies and practical clinical approaches, and devising potent tactics to improve immunotherapeutic outcomes.

## 2 TME overview and its characteristics

**Figure 1:**
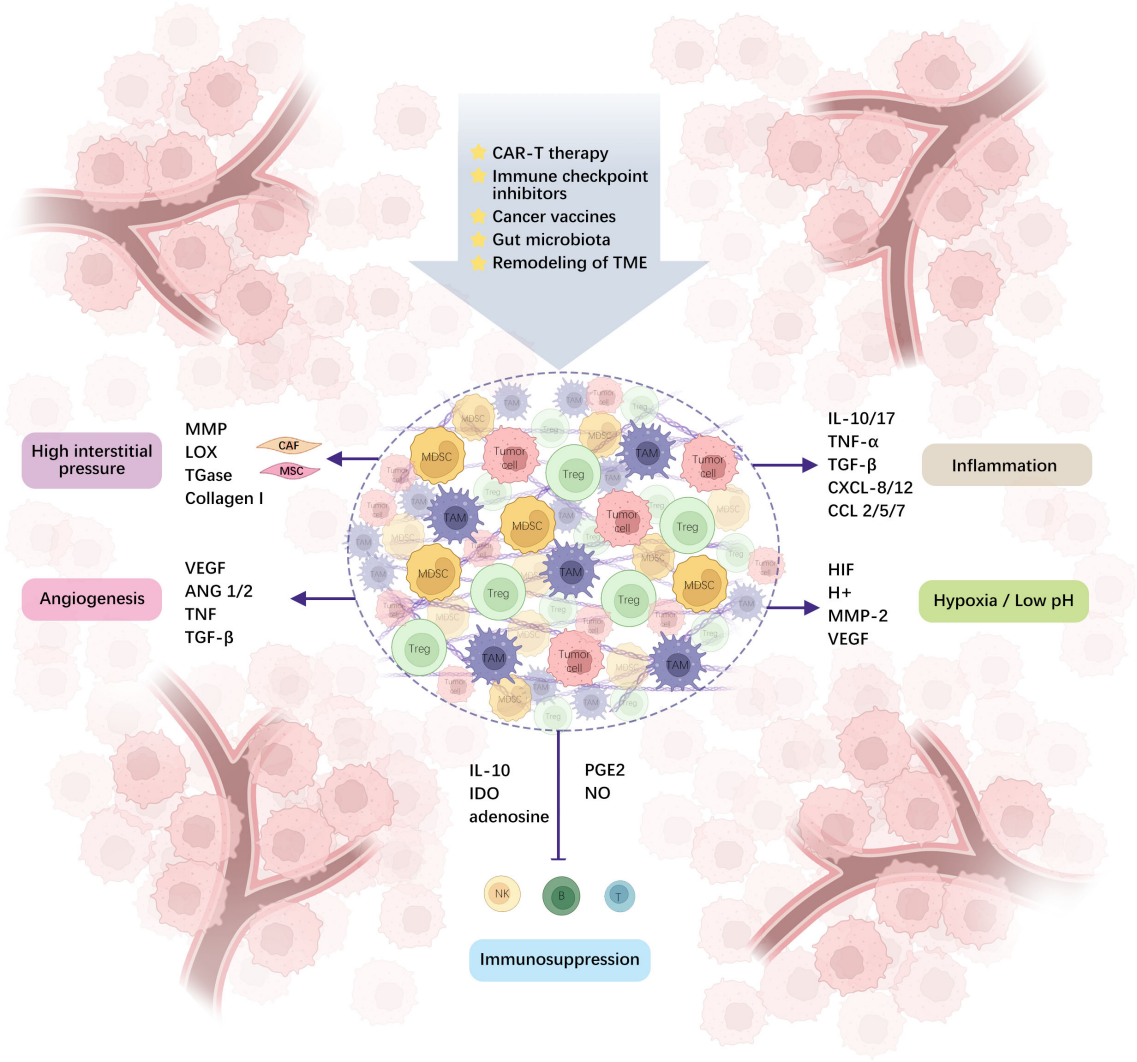
TME is comprised of different cells and molecules, and the interplay between immunosuppressive cells and tumor cells creates a microenvironment of hypoxia, acidity, high interstitial pressure, tumor-incapacitating angiogenesis, inflammation, and immunosuppression via diverse molecular pathways. (ANG1/2: angiopoietin-1 and 2, IDO: Indoleamine 2,3-dioxygenase, PGE2: prostaglandin E2, CAR-T: chimeric antigen receptor T-cell).

TME is a complex system made up of various cells and molecules that play a critical role in tumor progression and treatment resistance. Along with tumor cells, the TME includes endothelial cells, fibroblasts, and immune cells from both the innate and adaptive systems, whose early infiltration is vital for controlling tumors (5). Tumor cells interact with surrounding cells and structures within this environment to drive growth, invasion, and spread. Key features of the TME, such as hypoxia, low pH, high interstitial pressure, tumor vascular hyperpermeability, and inflammatory response, create conditions that support cancer cell survival while also making treatment more challenging (**Figure 1**). Furthermore, TME’s immunosuppressive nature, driven by cells like myeloid-derived suppressor cells (MDSCs), tumor-associated macrophages (TAMs), and regulatory T-cells (Tregs), limits the effectiveness of immunotherapy (**Figure 1**). These cells promote tumor development by secreting immunosuppressive molecules, such as transforming growth factor beta (TGF-β), or by modulating immune checkpoint molecules (6, 7). Understanding these intricate interactions is crucial for developing new therapeutic strategies.

### 2.1 Hypoxia

Hypoxia is a common characteristic of TME in various cancers. The rapid growth and high metabolic activity of tumor tissues increase the demand for oxygen and other energy substrates, such as glucose, leading to hypoxia. The tumor vasculature and hypoxic microenvironment act as complementary factors, with hypoxia stimulating the heightened production of erythropoietin and vascular endothelial growth factor (VEGF), promoting the growth of vascular endothelial cells in tumors. However, a disordered and chaotic arrangement within these blood vessels leads to their “non-functionality,” which exacerbates hypoxic conditions in the TME.

Hypoxia activates several adaptive pathways, including those involving hypoxia-inducible factor (HIF) and nuclear factor κB (NF-κB) (8). Hypoxic signals interact with additional cellular pathways to modify the malignant behavior of cancer cells, weaken the capabilities of cytotoxic T-lymphocytes (CTLs), and recruit Tregs, thereby reducing tumor immunogenicity and ultimately affecting cancer treatment outcomes. Under hypoxic conditions, HIF triggers the production of a range of immune-related elements. For instance, HIF-1α suppresses the function of CTLs and reduces the infiltrative capacity of immune cells by enhancing levels of molecules such as VEGF, PD-L1, and TGF-β (9, 10). Research has demonstrated that HIF-1α triggers increased expression of PD-L1 in cancer and myeloid cells. Bailey et al. demonstrated that targeting HIF-1α can enhance immune tolerance function associated with PD-1/PD-L1 checkpoints within normal tissues and eliminate immune escape mechanisms (11). Tian et al. showed that targeting the HIF-1α/STAT3/NF-κB signaling cascade reduces PD-L1 expression, boosts the proportion of CTLs, decreases Tregs, and diminishes immune suppression factors (12).

### 2.2 Low PH

The pH of the TME often decreases, resulting in an acidic environment. This is primarily due to inadequate oxygen supply, which compels cancer cells to rely on anaerobic glycolysis to meet their energy needs, consequently leading to lactate accumulation. Simultaneously, ion exchange proteins located on tumor cell membranes consistently move H+ ions from the intracellular environment to the extracellular space to prevent self-acidification. To varying degrees, these cellular responses contribute to TME acidification, resulting in an overall acidic environment. This low pH environment can inhibit cytotoxic reactions mediated by CD8^+^ T-lymphocytes and suppress the generation of interferon (IFN)-γ and other related cytokines by TH1 cells (13). Acidic conditions may increase the spread of cancer cells, thereby affecting their function in the immune system. Estrella et al. demonstrated that the administration of oral sodium bicarbonate is effective in increasing acidity levels around tumors, ultimately hindering tumor progression and preventing local spreading. This finding supports the theory that tumor invasion is influenced by acidity levels (14).

Lactate has different effects on immune cells, regardless of its pH-lowering properties. Lactate accumulation within TME has been demonstrated to inhibit CD8^+^ T cell function through multiple mechanisms. First and foremost, alterations in metabolic pathways represent the most significant impact. Lactate can modulate the intracellular pH of CD8^+^ T cells, thereby disrupting their metabolic processes, including glycolysis, oxidative phosphorylation, and the tricarboxylic acid (TCA) cycle (15, 16). This metabolic dysregulation leads to reduced Adenosine-5’-triphosphate (ATP) production, diminished effector function, and increased susceptibility to exhaustion. Furthermore, lactate induces the expression of inhibitory receptors such as PD-1 and CTLA-4 on T lymphocytes, which strengthens their interaction with corresponding ligands on tumor cells and Tregs (17). This interaction suppresses CD8^+^ T cell activity and facilitates tumor immune evasion. Studies have shown that lactate can modulate the epigenetic landscape of CD8^+^ T cells by influencing histone deacetylase (HDAC) and DNA methyltransferase activity (18). These epigenetic alterations inhibit the expression of genes critical for T cell activation and survival, further impairing their antitumor function. Another study revealed an unexpected immunoprotective role of lactate in antitumor immunity. It demonstrated that lactate, by inhibiting HDAC activity, enhances a subset of CD8^+^ T cells expressing stem-like TCF-1 (19). Lactate also influences the crosstalk between CD8^+^ T cells and other immune cells within TME. For instance, lactate promotes the differentiation of MDSCs and Tregs, while enhancing the expression of inhibitory molecules on their surfaces (17). The complex interplay of these immunosuppressive factors further diminishes CD8^+^ T cell functionality. Additionally, lactate enhances the activity of fibroblasts and myofibroblasts, leading to the production of extracellular matrix components that create a physical barrier, impeding T cell infiltration and function (20, 21). Researchers have shown that lactate can shift macrophages towards tolerogenic M2 types and modify the metabolism of Treg cells to sustain their functionality under glucose-deficient conditions. Therefore, tumor cells can evade elimination by starving effector T-cells of necessary nutrients. Additionally, they can aid the regulatory cell population by utilizing metabolic pathways, which may promote immune suppression in the TME (22). The inhibition of enzymes related to the glycolytic pathway due to the acidic microenvironment, particularly phosphofructokinase-1, leads to bidirectional interactions between tumor metabolism and the microenvironment pH (16). This bidirectional interaction further exacerbates the immunosuppressive state, thereby complicating the effectiveness of immunotherapy.

### 2.3 High interstitial pressure

Tumor-stromal fibrosis refers to the presence of abundant collagen fibers and other extracellular matrix components around the tumor, forming a state of high pressure. The stroma, a critical component of tissues and organs, consists of a variety of specialized connective tissue cells, such as fibroblasts, stromal cells, osteoblasts, and chondrocytes. These cells collaborate to provide structural support and maintain tissue integrity. The extracellular matrix (ECM) plays a crucial role in supporting cell structure and function within the stroma. The tumor stroma contributes to the TME by fostering treatment resistance, making it a significant factor in cancer therapy outcomes. Fibroblasts are the main cell type that produce collagen fibers and regulate collagen synthesis and degradation in the tumor stroma. Fibrosis restricts the spread and movement of tumor cells, thereby affecting tumor angiogenesis and oxygen supply. Studies have shown that the progression of breast tumors usually involves elevated accumulation, linking, and disrupted cleavage of type I collagen (23). Collagen restructuring occurs primarily because of the increased activity and expression of a range of enzymes (including lysyl oxidase (LOX), transglutaminase (TGase), and matrix metalloproteinase (MMP)) within the dynamic tumor matrix. Enhanced crosslinking of collagen and subsequent stiffening of tissues enhance the biomechanical responses of breast tissue, promoting the progression of breast tumors (24).

### 2.4 Tumor vascular hyperpermeability

**Figure 2:**
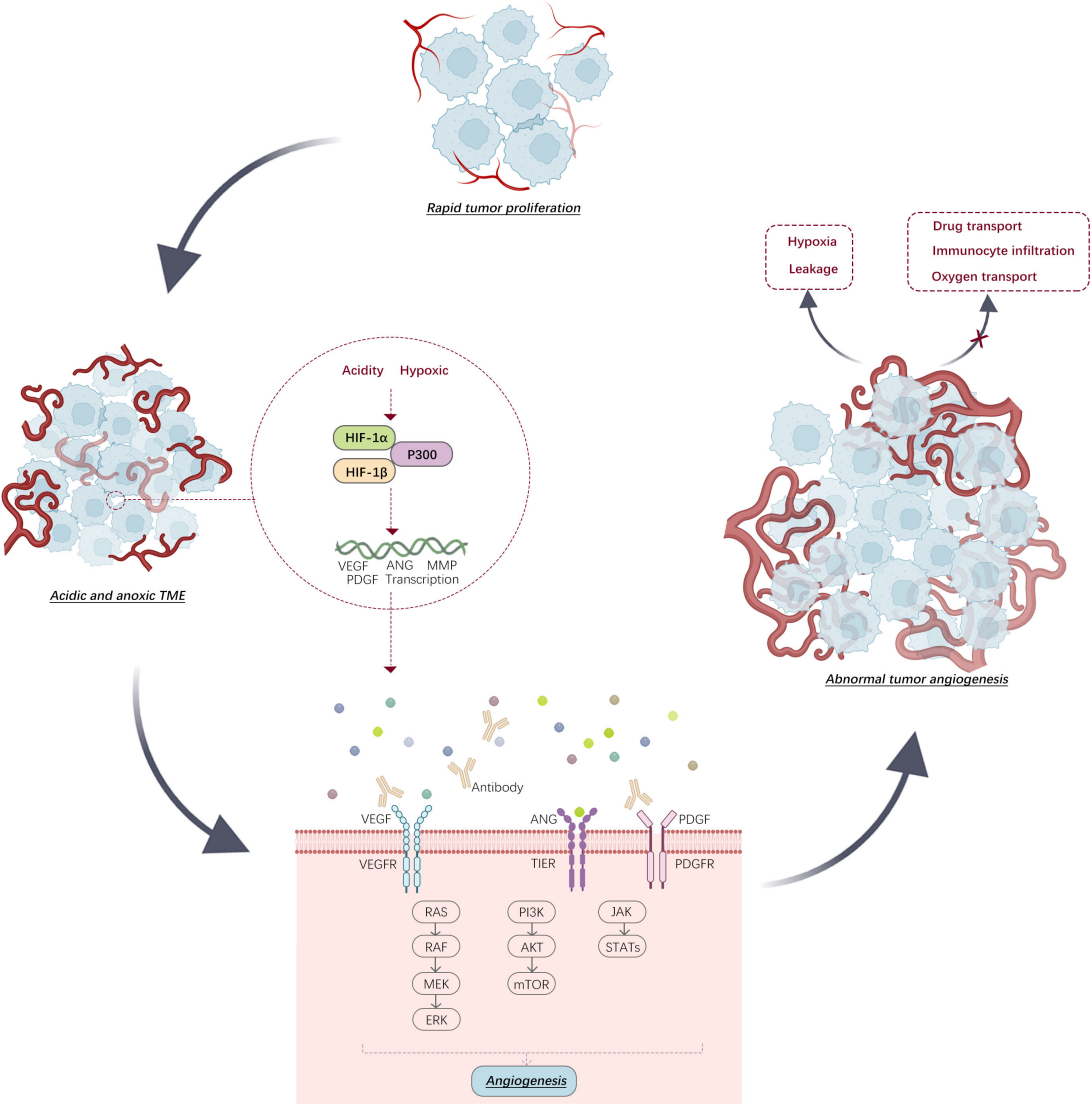
In environments that are both acidic and low in oxygen, transcription of angiogenic factors by HIF stimulates tumor angiogenesis. This progression can be hindered by specific antibodies. (VEGFR: vascular endothelial growth factor receptor, PDGFR: platelet-derived growth factor receptor).

In tumor cells, different transcription factors, such as HIF, can induce the production of pro-angiogenic factors, such as VEGF and platelet-derived growth factor (PDGF), thereby stimulating the *de novo* generation of blood vessels within the tumor and leading to the formation of a unique tumor vasculature, also referred to as the tumor-associated vasculature system (25) (**Figure 2**). The vascular system associated with tumors differs from that of healthy tissues in its complex, leaky, and disordered structure, which leads to the establishment of a drug-resistant TME (26). Cytokines such as tumor necrosis factor (TNF) and TGF-β are released by tumor cells. These cytokines affect endothelial cell adhesion and permeability, resulting in increased vascular permeability. Increased permeability results in tumor vessel leakage, which can impair tumor blood circulation and increase “intratumoral fluid pressure”. Anomalous circulation hinders drug transport and fosters tumor invasiveness, spread, immune suppression, inflammation, fibrosis, and resistance to treatment owing to oxygen deprivation (27).

### 2.5 Inflammatory response

During the development of tumors in hypoxic and acidic environments, both tumor tissues and adjacent tissue cells undergo extensive apoptosis, releasing cell fragments and chemotactic factors that attract inflammatory cells and trigger the release of inflammatory substances. Simultaneously, tumor growth induces an immune reaction, causing inflammatory cells to accumulate in the area and provoke a strong inflammatory reaction. Inflammation is a basic innate immune response that disrupts tissue homeostasis but can have a dual effect in promoting cancer (28). In the TME, inflammatory responses mainly occur via the activation of immune cells and the generation of inflammatory mediators. In the context of tumor initiation and progression, inflammatory mediators act more like a balance between anti-tumor and pro-tumor inflammatory mediators, dividing tumor immune editing into three distinct phases: elimination, balance, and evasion. Tumor-associated dendritic cells, MDSCs, tumor-associated macrophages (TAMs), Th17 cells, Tregs, and cancer cells induce immune suppression by secreting various immunosuppressive cytokines and molecules (2). Among these, TAMs, the primary type of inflammatory cells in the TME, sustain significant inflammation. TAMs induce a shift from M1 to M2 polarization by upregulating inflammatory molecules (e.g., IL-10, TGF-β) and secreting chemokine components (e.g., CCL2, CCL5, CCL7, CXCL8, CXCL12) to maintain an immune suppressive phenotype. In a study on colon cancer, it was reported that during the initial stages of inflammation-associated colon cancer, bacterial lipopolysaccharide (LPS) regulates the expression of CCL2 in colonic epithelial cells, which in turn regulates the accumulation of monocyte-like macrophages (MLMs). Furthermore, this stimulation increases the production of IL-1β by MLMs, inducing Th cells to produce IL-17 and promote inflammation, driving the onset of cancer (29).

Recent reports (30) support the association between chronic inflammation, IL-6 cytokine signaling, and resistance to immunotherapy. IL-6 and related cytokines are critical in the interplay between inflammation and cancer. Within TME, IL-6 and its family members promote tumor cell proliferation, survival, and angiogenesis by activating signaling pathways such as JAK/STAT, PI3K/AKT, and MAPK. IL-6 induces the expression of anti-apoptotic proteins, such as Bcl-2 and BCL-XL, in cancer cells, thereby enhancing their resistance to apoptosis. The IL-6 cytokine family also exerts profound effects on immune cells, promoting the differentiation of T cells into Th17 cells, which secrete pro-inflammatory cytokines that further support tumor growth (31). Hailemichael et al. revealed in cancer models receiving ICIs therapy that IL-6R inhibition alleviates ICI-related toxicity by reducing Th17 differentiation, pro-inflammatory cytokine secretion, and neutrophil chemokine production, while simultaneously enhancing tumor immunity by increasing Th1 and CD8^+^ T effector cells (32). Beyond IL-6, other members of the IL-6 cytokine family, such as IL-11, leukemia inhibitory factor (LIF), and oncostatin M (OSM), also play critical roles in the interplay between inflammation and cancer (31). For instance, IL-11 exhibits potent pro-tumorigenic effects in gastric and colorectal cancers. In a study on hepatocellular carcinoma (HCC), IL-11 was shown to promote tumor cell proliferation and resistance to apoptosis by activating the STAT3 signaling pathway, contributing to cancer recurrence in postoperative HCC patients (33). Given the multifaceted roles of the IL-6 cytokine family in cancer progression and immunosuppression, various strategies have been developed to target this cytokine family in cancer immunotherapy. One primary approach is the use of monoclonal antibodies (mAbs) that directly target IL-6 or its receptor components. Tocilizumab, an IL-6 receptor antagonist, and Siltuximab, an anti-IL-6 antibody, have been shown to inhibit IL-6 signaling; they were initially studied for treating cytokine release syndrome, and clinical and preclinical studies are now underway to evaluate their efficacy in a variety of cancers (34, 35). Another approach involves the development of small molecule inhibitors targeting IL-6-activated signaling pathways. For example, JAK inhibitors like Ruxolitinib and Baricitinib have been used to block the JAK/STAT pathway, a key conduit for IL-6 signal transduction. These inhibitors have demonstrated efficacy in treating certain hematologic malignancies, and their potential is currently being explored in solid tumors (**Table 1**).

**Table 1:** Drugs targeting TME in clinical studies.

Strategies	National Clinical Trial number	Drugs	Status	Cancer types	Mechanisms	Ref.
Improving hypoxic	NCT03401788 NCT02974738 NCT03634540	Belzutifan	Phase I/II	Renal Cell Carcinoma	HIF-2α inhibitor	(150–152)
Modulating the acidic microenvironment	NCT01791595	AZD3965	Phase I	Advanced solid tumors, lymphoma	Monocarboxylate Transporter 1 Inhibitor	(153)
Regulating tumor angiogenesis	NCT04322539	Fruquintinib	Phase III	Colorectal cancer	VEGFR1 antagonist	(154)
	NCT04724239 NCT02715531 NCT02759614 NCT04737187	Bevacizumab	Phase I/II/III	Colorectal cancer, hepatocellular carcinoma, non-small-cell lung cancer	VEGF-A inhibitor	(155–158)
	NCT04900363 NCT04047290	Ivonescimab	Phase I	Advanced Solid Tumors, non-small-cell lung	PD-1/VEGF-A bispecific antibody	(76, 77)
	NCT02898077	Ramucirumab	Phase III	Gastric or gastroesophageal junction adenocarcinoma	VEGFR2 antagonist	(159)
	NCT02597036	LY3127804	Phase I	Advanced Solid Tumors	ANG2 inhibitor	(160)
	NCT01609790	Trebananib	Phase II	Glioblastoma	ANG1/2-Tie2 inhibitor	(161)
Targeting CAFs	NCT01722149	FAP-specific re-directed T cells	Phase I	Fibroblast activation protein-positive malignant pleural mesothelioma	Targeting FAP	(162)
	NCT05410821	177Lu-LNC1004	Phase I	Metastatic Radioiodine-Refractory Thyroid Cancer	Targeting FAP	(163)
	NCT01722149	CART-FAP	Phase I	Pleural mesothelioma	Targeting FAP	(164)
Targeting TAMs	NCT02323191	Emactuzumab	Phase I	Advanced solid tumor	CSF1R antibody	(165)
	NCT04691375	PY314	Phase I	Advanced renal cell carcinoma	Targeting TREM2	(166)
	NCT02637531	IPI-549	Phase I	Advanced Solid Tumors	PI3K-γ inhibitor	(167)
Targeting MDSCs	NCT02826486	BL-8040	Phase II	Pancreatic cancer	CXCR4 antagonist	(168)
	NCT02936752	Entinostat	Phase I	Myelodysplastic syndromes/neoplasms, acute myeloid leukemia	Histone deacetylase inhibitors	(169)
	NCT01688999	Cabozantinib	Phase II	Metastatic urothelial carcinoma	Multikinase inhibitor of MET, VEGFR, AXL, and RET	(170)
Targeting Tregs	NCT01929486	KW-0761	Phase I	Advanced Solid Tumors	Anti-CCR4 antibody	(171)
	NCT04504669	AZD8701	Phase I	Anaplastic cell lymphoma	FOXP3 inhibitors	(172)
	NCT02983045 NCT03635983	NKTR-214	Phase I/II/III	Advanced Solid Tumors	Selective activation of IL-2R	(173–175)
	NCT02923349	INCAGN01949	Phase I/II	Colorectal, ovarian, non-small cell lung cancers	Human immunoglobulin G1κ anti-OX40 agonist monoclonal antibody	(176)
Targeting IL-6/IL-6R	NCT01024036 NCT01484275	Siltuximab	Phase II	Lymphoproliferative disorder, multiple myeloma	IL-6 blocker	(177, 178)
	NCT04258150	Tocilizumab	Phase I/II	Pancreatic cancer	IL-6R blocker	(116)
	NCT02092324 NCT02974647 NCT02713386	Ruxolitinib	Phase II	Chronic neutrophilic leukemia, atypical chronic myeloid leukemia, T-cell lymphomas, advanced ovarian cancer	JAK inhibitor	(179–181)
Targeting TGF-β	NCT02947165	NIS793	Phase I/II	Advanced solid tumors	anti-TGF-β monoclonal antibody	(182)
	NCT02517398 NCT03833661	Bintrafusp alfa	Phase I/II	Advanced non-small cell lung cancer, biliary tract cancers	a bifunctional fusion protein targeting TGF-β and PD-L1	(183, 184)
	NCT02688712 NCT02423343	Galunisertib	Phase I/II	Advanced rectal cancer, non-small cell lung cancer	TGF-β1 receptor type I inhibitor	(185, 186)

### 2.6 Immunosuppressive nature

Within the TME, interactions occur between cancer and stromal cells, which function together to enhance tumor growth and spread. The rise in immunosuppressive cells and molecules, inhibits antitumor immunocyte activity and creates conditions enabling immune evasion.

MDSCs are a collection of highly immunosuppressive cells whose main function is to inhibit immune reactions and shield tumor cells from attack (36). This cluster of highly immature bone marrow-derived immune cells primarily includes polymorphonuclear and monocytic MDSCs (37). MDSCs suppress immune cell activity via cell-to-cell contact mechanisms involving interactions between surface molecules and receptors. Additionally, MDSCs are capable of imparting immunosuppressive properties by generating extracellular factors such as nitric oxide (NO), opioid-like peptides, TGF-β, and IL-10. The surface receptor TIM-3 on MDSCs binds to T-lymphocytes and upregulates PD-L1 expression, thus inhibiting T-lymphocyte functionality. MDSCs promote angiogenesis and the development of pre-metastatic niches through mechanisms unrelated to immunity. They achieve this through the secretion of increased levels of VEGF and basic fibroblast growth factor (bFGF). These factors significantly affect the reshaping of the TME, thereby facilitating tumor progression (38, 39).

TAMs predominantly exhibit an M2 phenotype and are strongly correlated with unfavorable patient outcomes. Hypoxic and highly lactate-rich TMEs promote inflammation-suppressive polarization. A study focusing on tumor acidosis elucidated that TAMs sense the acidic environment, leading to high cAMP expression and subsequent induction of the transcription factor ICE, resulting in the polarization of TAMs towards a pro-tumor phenotype (40). High levels of AMP bind to adenosine receptors, such as A2AR and A2BR, on the surface of macrophages, promoting M2 polarization (41). Tumor-derived prostaglandin E2 (42), fatty acids (43), and α-ketoglutarate (44) are among the various agents that induce M2 polarization of macrophages, promoting a pro-tumor phenotype.

In oxygen-deprived TMEs, Tregs contribute to tumor progression while simultaneously hindering antitumor immunity. Treg cells engage with immune cells such as CD4^+^ Th and CD8^+^ CTLs, inhibiting their activity and function by binding to surface markers such as cytotoxic T-lymphocyte-associated protein 4 (CTLA-4) and PD-1. Tregs undergoing apoptosis release elevated amounts of ATP, which, in the presence of CD39 and CD73, degrades into adenosine and exerts immunosuppressive effects through A2A receptors on T-cells. Tregs release related factors such as TGF-β and IL-10/35, downregulating anti-tumor immunity and inhibiting antigen presentation by dendritic cells (DCs), further dampening immune responses by lymphocytes (45). In patients with cancer, a poor prognosis is often linked to the heightened presence of FoxP3+ Tregs within tumors. This correlation provides a solid foundation for exploring Treg targeting in cancer immunotherapy (46).

TGF-β, secreted by tumor cells, cancer-associated fibroblasts, and immunosuppressive cells, mediates tumorigenesis and progression through engagement with the TGF-β receptor (TGF-βR) family, activation of Smad transcription factors, and subsequent regulation of downstream gene expression (47). Among the TGF-β family members, the TGF-β1 isomerase exerts the most profound influence. Studies have elucidated that TGF-β1 participates in immunosuppression via multiple mechanisms, creating a favorable milieu for tumor growth and dissemination (48). Upon binding to the TGF-βR, TGF-β1 activates receptor-activated Smads (R-Smads), predominantly Smad2 and Smad3. These R-Smads form complexes with the common Smad (Co-Smad, Smad4), translocate to the nucleus, and modulate the expression of specific genes, including various factors pertinent to effector T cell functionality, such as IFN-γ and TNF-α. TGF-β1 downregulates the expression of these cytokines, diminishing the cytotoxicity and antitumor capabilities of effector T cells (49). TGF-β1 also suppresses the differentiation of Th1 and Th2 cells by downregulating the expression of T-bet and GATA3 through Smad-dependent pathways. Moreover, TGF-β1 modulates T cell activation and function through Smad-independent signaling pathways, including the MAPK, PI3K/Akt, and Rho GTPase pathways (50). Within TME, TGF-β1 potentiates the differentiation of immunosuppressive cells, thereby enhancing the immunosuppressive status of the microenvironment. It has been shown that TGF-β1 predominantly upregulates Foxp3 expression through the classical Smad-dependent pathway, promoting the differentiation of Tregs (50). TGF-β1 also facilitates the polarization of M2-type TAMs, further exacerbating immunosuppression (51). Recent evidence suggests that TGF-β1 activates the Smad2/3 complex to upregulate the expression of SOX18. Overexpression of SOX18, in turn, increases the transcription levels of PD-L1 and CXCL12, promoting the accumulation of immunosuppressive TAMs and Tregs within the microenvironment, thereby exacerbating the progression of hepatocellular carcinoma (52). In another study focusing on acute myeloid leukemia (AML), Wang et al. discovered that Glycoprotein A Repetitions Predominant (GARP), expressed on the surface of Tregs, can activate and release bioactive TGF-β1. This active TGF-β1 can induce dysfunction in natural killer (NK) cells of AML patients and facilitate early AML relapse (53). Additionally, TGF-β1 directly impairs the antigen-presenting ability of DCs by downregulating the expression of co-stimulatory molecules on DCs (such as CD80 and CD86) and by reducing the expression of major histocompatibility complex (MHC) class I molecules or influencing antigen processing (54). Increasing evidence indicates that TGF-β1 upregulates the expression of exhaustion markers such as PD-1, TIM-3, and LAG-3, driving effector T cells into a state of exhaustion (55, 56). These immunosuppressive effects synergize to afford TGF-β1 a formidable role in promoting tumorigenesis within the TME. Targeted interventions against the TGF-β1 signaling pathway are considered a pivotal strategy to augment antitumor immune responses, demonstrating certain promise in preclinical and clinical investigations (53, 57) (**Table 1**).

## 3 Immunotherapeutic strategies targeting the TME

The TME, as a crucial milieu for tumor growth and development, with hypoxic and acidic features, along with the presence of immunosuppressive cells and tumor vasculature, collectively creates a “pro-tumor, anti-immune” microenvironment. Therefore, targeting immunosuppressive components within the TME has emerged as a potent and effective strategy for reshaping the TME.

### 3.1 Improvement of hypoxic environment

Hypoxia is frequently observed in the TME, whereby a hypoxic environment can alter the metabolism of cancer cells and enhance their potential for invasion and metastasis while rendering tumors resistant to standard treatment modalities such as radiotherapy and chemotherapy. Strategies to increase oxygen delivery by pharmacological vasodilation to increase blood flow to the tumor have been proposed, but the non-functional nature of the tumor vasculature makes it challenging to improve blood flow to tumor tissues in practice (58). In recent years, researchers have explored methods for treating tumors in hypoxic environments.

Multiple studies have indicated that inhibition of HIF and its subsequent target genes is a successful method. Salman et al. demonstrated that the small molecule 32-134D successfully hindered gene activation controlled by HIF-1/2 in HCC in a mouse model. This action halts the growth of human and murine HCC tumors, increasing the elimination rate from 25% to 67% (59). Other research showed that honokiol (HNK) downregulates levels of HIF-1α and its associated factors (such as PDK1, HK2, and GLUT1), inhibiting HIF-1α-dependent glycolysis and inducing cancer cell death through apoptosis (60). Yan et al. demonstrated that HIF-2α is essential for enhancing chemical resistance in breast cancer cells exposed to low levels of oxygen. They identified the specific pathway through which HIF-2α influences this resistance, involving SOD2, mtROS, PDI/GRP78, and the unfolded protein response (UPR). This suggests that developing inhibitors targeting HIF-2α could represent a potentially effective strategy to combat chemoresistance in hypoxic breast cancer (61). Li et al. used RNA-seq for whole transcriptome analysis and intercellular space analysis to discover that P4HA2 stabilizes HIF-1α protein to activate genes downstream, thereby resulting in decreased levels of ROS in bladder cancer cells. This ultimately leads to resistance to erdafitinib. The discovery of P4HA2 provides a potential and valuable target for targeted therapy of acquired resistance in bladder cancer (62). CAR-T-cell therapy is a promising form of immunotherapy for combating cancer, given its limitations in solid tumors. Researchers have developed various combination therapies to enhance their efficacy. High levels of HIF-1α expression in individuals with cervical cancer are associated with unfavorable long-term outcomes. However, a recent study reported that PX-478, an inhibitor of HIF-1α, significantly degrades the cytotoxic activity of mesothelin-CAR-T-cells (meso-CAR-T-cells) targeting cervical cancer cells and induces T-cell exhaustion (63). Nevertheless, Luo et al. showed that when PX-478 is used in combination with ICIs, it promotes T-cell-induced apoptosis of tumor cells. Furthermore, in mice treated with both PX-478 and anti-PD-1 antibodies, researchers have observed a marked slowing of tumor development along with increased survival rates (64).

Tumor autophagy is a key biological process within the extensive regulatory network of HIFs. HIFs promote autophagy by regulating downstream target genes, including autophagy-related genes. Autophagy activation can further stabilize HIF proteins, creating a positive feedback circuit that contributes to tumor resistance (9). Research by Li et al. revealed that the N6-methyladenosine (m6A) reader protein induced by HIF-1α stimulates the expression of autophagy-related genes *ATG2A* and *ATG14* in a manner sensitive to m6A, which is linked to hypoxia-induced autophagy and the advancement of HCC (65). In another study, under hypoxic conditions, HIF-1α was shown to induce upregulation of the tumor cell *MALAT1*, further promoting the growth and spread of triple-negative breast cancer (TNBC) cells by autophagy activation. Conversely, inhibition of autophagy in TNBC cells under anoxic conditions can be achieved by targeting *MALAT1* with mirR141-3p. These findings indicate a significant interaction between HIF-1α, Malat1, and miR-141 in tumor progression (66). Several studies have shown that targeting HIF suppresses tumor growth (**Table 1**). However, the interactions of combination therapies within the complex TME are worth considering, and the mechanisms underlying the interactions between HIF inhibitors and various immunotherapies warrant further investigation.

In addition to targeted therapies to improve the hypoxic environment, preclinical models have been used to study physical approaches to alleviate hypoxia. In a lung cancer mouse model, exposure to additional oxygen (in breathing O_2_, levels rose from 21% to 60%) demonstrated that exogenous oxygen supplementation can reduce HIF-1α levels along with its downstream proteins, reversing the hypoxic, adenosine-rich TME, thereby promoting tumor regression (67). However, the risks involved in high-dose oxygen supply must be considered in clinical applications. Another similar method is hyperbaric oxygen (HBO) therapy, which is frequently employed as a supplementary treatment for a range of pathological conditions but has not yet been approved for cancer treatment. However, as an adjunctive therapy, HBO has demonstrated capabilities in restraining tumor progression. For example, research by Liu et al. indicated that HBO promotes the distribution of PD-1 antibodies and penetration of T-cells into tumor tissues by reducing the primary constituents of the ECM, disrupting hypoxia-induced immune suppression (68). Owing to the abnormal vascularity of tumor tissues, improving tumor tissue oxygen levels through physical means has inherent limitations. In recent years, researchers have focused on developing a new type of delivery system that accelerates the breakdown of internal H_2_O_2_ to produce O_2_ in tumor tissues. Yao et al. encapsulated catalase and a HIF-1 blocker within the aqueous compartments of liposomes to alleviate the hypoxic state in tumors. This approach enhances the sensitivity of iodine-125 brachytherapy and achieves the eradication of esophageal cancer (69).

### 3.2 Modulation of acidic TME

The acidic TME can drive cancer cells to migrate toward blood vessels while hindering the action of immune cells. However, restoring the acidic environment can reverse this immune suppression by enhancing the effectiveness of checkpoint inhibitor therapy targeting PD-1 antibodies (70). Lactic acid production, mediated by the enzyme lactate dehydrogenase (LDH), is a primary factor that leads to an acidic TME. Additionally, tumor vascular integrity, changes in the ECM, inflammatory responses, cell apoptosis, expression of ion-exchange proteins, and factors released by tumor cells collectively result in an acidic TME.

Currently, multiple studies are dedicated to overcoming the low pH characteristic of the TME to inhibit cancer cell growth and suppress immune-related cells. The most promising strategy is to therapeutically target major pathways involved in lactic acid production by inhibiting glycolysis. Zhang et al. used vesicular nanoparticles that contained cationic lipids to decrease the expression of LDH A (LDHA) in cancer cells. This causes a shift in acetate metabolism, a decrease in lactate levels, and the stabilization of the acidic TME. This therapy increases the migration of CD8^+^ T-lymphocytes and NK cells, reduces the quantity of immunosuppressive T-lymphocytes, and effectively suppresses cancer progression in immunologically active melanoma and breast tumor models (70). Another preclinical model of Ewing’s sarcoma demonstrated that inhibiting LDH to target glycolysis can suppress tumor growth (71). Another approach for targeting lactic acid is to inhibit monocarboxylate transport proteins (MCT1 and MCT4). Currently, MCT1 inhibitors (e.g., AZD3965) have successfully entered clinical trials (**Table 1**). Relevant *ex vivo* experiments have shown that simultaneously blocking MCT1 and MCT4 significantly prolongs overall survival in cancer models (72). Based on advancements in nanobiotechnology, Li et al. developed a stratified fibrous device for localized drug administration. The fibrous framework incorporates proton-pump inhibitors that are steadily released to impede the outflow of hydrogen ions from inside tumor cells. Experiments conducted inside living organisms revealed that this localized drug administration device renewed pH levels in the TME from 6.8 to 7.2, demonstrating remarkable immunotherapeutic effects (73). Although numerous strategies have been tested, whether they can be used in clinical settings remains unclear and requires further exploration.

### 3.3 Regulation of tumor angiogenesis

The “abnormalization” of tumor blood vessels is a crucial factor in leading to the low oxygen and acidic TME, making the normalization of tumor vasculature crucial for cancer therapy. Researchers have proposed various strategies to enhance the efficacy of immunotherapy targeting abnormal tumor vasculature. One strategy involves utilizing anti-angiogenic medications, such as VEGF blockers, to reduce tumor vasculature formation and increase vascular permeability (**Figure 2**). Bevacizumab, an anti-VEGF antibody, is a commonly used antiangiogenic medication. Combination therapy of bevacizumab with other immunotherapies has demonstrated clinically significant overall survival benefits in certain cancers, such as mesothelioma and advanced colorectal cancer (**Table 1**).

Another strategy involves combining anti-angiogenic agents with additional treatments, including ICIs and adoptive cell therapy (ACT). A clinical study targeting TNBC reported that combination therapy with low-dose VEGFR2 inhibition and anti-PD-1 therapy showed excellent tolerability and efficacy. Minimal inhibition of VEGFR2 leads to enhanced infiltration of immunocytes, promoting CD8^+^ T-lymphocytes to secrete osteopontin, which in turn induces tumor cells to produce TGF-β, upregulating PD-1 levels in immunocytes and thereby improving the effectiveness of ICIs (74). Abnormal tumor vasculature restricts the entry of immune cells into the tumor interior, thereby affecting the efficacy of immunotherapy. Shrimali et al. demonstrated that by disrupting the VEGF/VEGFR-2 axis with anti-VEGF antibodies to “normalize” the tumor vascular system and using it in conjunction with ACT, antibody therapy significantly promotes immune cell infiltration. Furthermore, this combination therapy significantly inhibited the growth of vascularized melanoma tumors (P = 0.009) and improved mouse survival rates (P = 0.003) (75). Ivonescimab, a recently authorized bi-specific antibody intended for the treatment of advanced solid malignancies, targets VEGF and PD-1. Data from phase I trials have demonstrated significant clinical advantages (76, 77). Currently, dozens of anti-angiogenic drugs that block the VEGF signaling pathway have been authorized for cancer therapy. Various antiangiogenic agents targeting ANG1, ANG2, and the tyrosine kinase receptor TIE2 pathway are currently undergoing clinical trials (**Table 1**). Furthermore, studies have demonstrated that anti-VEGF antibodies can prevent TAMs from differentiating into an M2-like phenotype and eliminate VEGF-induced DC maturation inhibition (78).

### 3.4 Targeting of substrates

Cancer-associated fibroblasts (CAFs) are the predominant stromal cells in tumor tissues. In certain types of cancer, the content of CAFs is inversely correlated with treatment outcomes in patients with cancer undergoing chemotherapy and immunotherapy (79). This is because anti-cancer drugs are easily restricted by stromal barriers; therefore, regulating CAFs could provide an effective auxiliary method to enhance sensitivity to anti-cancer drugs (80).

Fibroblast activation protein (FAP) is specifically expressed on proliferating fibroblasts, and its presence on the surface of CAFs can be detected in many types of cancer with poor prognoses. FAP is increasingly viewed as a promising target for therapy and molecular imaging applications (81). Considering the widely used CAR-T therapy, the integration of FAP expression with CAR-T-cells appears to offer a potentially effective treatment approach. Schuberth et al. were the first to show that ACT therapy targeting FAP effectively suppresses the proliferation of FAP-expressing cancer cells within the peritoneal cavity in murine models. This study underscores the promising capability of FAP CAR-T-cell therapy to specifically eliminate malignant cells that overexpress FAP (82). Because of the carcinogenic properties of FAP-positive CAFs, it can be reasonably assumed that employing CAR-T-cells directed at FAP may enhance patient prognosis. This was confirmed using an orthotopic TNBC mouse model. Das et al. used engineered FAP UCAR-T-cells to target CAFs, and their results showed significant efficacy in depleting CAFs and reducing fibrosis. Additionally, the co-administration of ACT therapy and anti-PD-1 checkpoint inhibitors resulted in a noteworthy reduction in tumor size and prolonged the overall survival of mice (83). In a study concerning pancreatic cancer, FAP CAR-T-cell therapy blocked the infiltration of MDSCs and increased the persistence of T-lymphocytes (84). Hence, targeting FAP could represent an approach for reshaping the TME and preventing immune escape. Related preclinical T-cell studies and clinical trials are ongoing (**Table 1**).

In comparison with the systemic targeting of FAP therapy, Zhen et al. described nanoparticle-based photoimmunotherapy (nano-PIT) (85). This strategy conjugates FAP single-chain fragment variable (scFv) to the surface of ferritin, and when exposed to light, nano-PIT can efficiently eradicate CAFs within tumors, further inhibiting the secretion of CXCL12 and ECM deposition. This significantly enhances T-cell infiltration and inhibits tumor growth. Other approaches that target FAP include antibodies that inhibit the proteolytic activity of FAP and FAP-targeted vaccines (86, 87).

During cancer development, normal fibroblasts are stimulated by various cytokines to differentiate into CAFs. The main cytokines involved in this process include TGF-β, bFGF, and IL-1β. Research indicates that this activation is largely driven by TGF-β (88). Therefore, by exploiting this mechanism, researchers have designed another approach to eliminating CAFs by targeting TGF-β. Yang et al. constructed a nanodrug delivery system (HA-LSL/siTGF-β) designed for the effective transport of siRNA aimed at TGF-β to simultaneously silence TGF-β in stromal and cancer cells, reducing stromal cell deposition and promoting nanoparticle penetration. The findings of that study verified that this strategy improved oxygenation and the effective delivery of ICIs. Additionally, a reduction in the percentage of immunosuppressive cells such as Tregs and MDSCs has been observed (89). Pei and colleagues introduced an innovative strategy for sequential targeting that initially targets stromal TGF-β signaling, reverses CAF activation, and weakens the dense stromal barrier to facilitate subsequent drug delivery. These findings indicate that, in contrast to the use of gemcitabine alone, this sequential targeting method significantly extended the survival of mice affected by pancreatic cancer (90).

### 3.5 Cell-based immunotherapy

Cellular therapy, an emerging modality within the realm of tumor immunotherapy, primarily aims to modulate and augment the patient’s intrinsic immune response against tumor cells, thereby achieving the goal of tumor eradication. In recent years, researchers have been diligently working on the development of various cellular therapeutic strategies, including CAR-T cells, CAR-NK cells, TCR-engineered T cells, and tumor-infiltrating lymphocytes (TILs), to target the intricate biological characteristics of TME.

#### 3.5.1 CAR-T-cell therapy

CAR-T-cell therapy represents an innovative immunotherapeutic strategy that entails the genetic modification of patients’ lymphocytes to express CARs directed against tumor-associated antigens. CAR design integrates domains that bind to antigens with those that activate CTLs, enabling CAR-T-cells to identify and destroy cancerous cells (91). Using gene editing techniques, T-cells are endowed with a scFv, which is formed by connecting the light chain (VL) and heavy chain (VH) from a monoclonal antibody through peptide linkage, retaining antibody specificity and affinity for the cognate antigen. Upon identifying the tumor antigen, downstream co-stimulatory signaling molecules activate the T-cells, which further release cytokines such as IL-2 and IFN-γ, thereby inducing apoptosis of target cells (92). CAR-T-cells binding to target antigens through scFv offers species-specific recognition that is independent of MHC expression, avoiding tumor escape through MHC regulation and conferring the capability of CAR-T-cells to identify non-peptide antigens (93). By altering the receptor specificity of CAR-T-cells, the immunosuppressive components can be effectively identified, eliminating immune-suppressive components.

Studies have shown that targeting F4/80 (a macrophage marker) by CAR-T-cells can deplete TAMs and improve survival outcomes in animal models of ovarian and pancreatic cancers (94). Rodriguez-Garcia et al. demonstrated that TAM subsets expressing folate receptor β (FRβ) exhibit immune-suppressive M2-like characteristics. Meanwhile, eliminating FRβ-expressing TAMs within the TME by CAR-T-cells brings about the infiltration of CD8^+^ T-lymphocytes (95). Additionally, Li et al. conducted *in vitro* experiments showing that three types of innate T-lymphocytes (including mucosal-associated invariant T (MAIT) cells, invariant NK T (iNKT) cells, and gamma delta T (γδT) cells) possess significant TAM-killing capabilities and verified the heightened anticancer abilities of CAR-engineered innate T-lymphocytes (96). Because CCR4 is mainly expressed by Th2/17 cells and Tregs, Watanabe et al. developed anti-CCR4 CAR T-cells and demonstrated their capacity to selectively deplete Tregs. They successfully induced long-term remission and excellent anti-cancer outcomes in an animal model of human T-cell transplantation (97). Zannikou et al. demonstrated that MDSCs in the TME of glioblastomas express IL-15Rα. Utilizing CAR-T-cells engineered with IL-15 offers a two-pronged approach for targeting both cancer cells and the MDSCs found in glioblastomas. This approach enhances the recruitment of CD8^+^ T-lymphocytes, B-lymphocytes, and NK cells to the tumor site (98). Combination therapy has become a mainstream approach because of the complexity of the TME. Wu et al. analyzed the collective effects of GPC3-CAR T-cells and sorafenib in HCC models with normal immune function and immune deficiency. The results revealed that sorafenib boosts the anti-cancer effect of CAR-T-cells by stimulating IL-12 secretion by TAMs and triggering apoptosis of cancer cells (99). A similar conclusion was confirmed in another study in which lenvatinib upregulated the levels of CXCL10 and CCL8 in tumors, thereby boosting CAR-T-cell infiltration and decreasing immune escape (100).

Within the TME in multiple myeloma, high concentrations of TGF-β lead to an immunosuppressive environment. To address this issue, Alabanza et al. developed a unique B-cell maturation antigen (BCMA) CAR. This CAR includes a dominant-negative TGF-β receptor II (DNR II), which lacks the kinase structural domain. This DNR II confers T-cells with the ability to resist the suppressive characteristics of TGF-β, demonstrating improved anti-tumor cytotoxicity in a mouse tumor model overexpressing TGF-β (57). The same modification has demonstrated enhanced anti-cancer outcomes in prostate (101) and ovarian cancer (102) models. Recent research has demonstrated that SMAD7 (a TGF-β signaling inhibitor) can eliminate communication between TGF-β and NF-κB signaling pathways by downregulating TGF-βRI. Constructing a CAR structure on engineered T-cells that co-express SMAD7 and HER2 shows resistance to TGF-β-induced T-cell exhaustion (103). Separate research has indicated that utilizing CRISPR/Cas9 gene editing technology to eliminate endogenous TGF-βRII can decrease Treg conversion and inhibit the exhaustion of CAR-T-cells (104).

Recent findings suggest that immune-inhibitory cell-derived IL-10 reduces lymphocyte infiltration by upregulating the expression of PD-L1 in monocytes (105). This has been confirmed in glioblastoma (106), bladder cancer (107), and drug-resistant multiple myeloma (108), thus establishing a foundation for the surveillance and targeting of IL-10. Sullivan et al. disrupted IL-10 signaling using IL-10 receptor-blocking antibodies, which reversed immune suppression mediated by myeloid cells (109). Given the characteristic overexpression of IL-10R in most AML cells, Chen et al. developed a new ligand-driven CAR-T-cell model focused on IL-10R, which demonstrated excellent cell-killing abilities against AML cells in laboratory and animal models. However, this study revealed the potential risks involved in reshaping the suppressive TME by boosting IL-10 signaling (110). Contrary to the notion that IL-10 promotes immunosuppressive TMEs, it activates tumor-resident CD8^+^ T-lymphocytes (111). Guo et al. revealed that boosting oxidative phosphorylation by extending the half-life of an IL-10-Fc fusion protein could effectively strengthen the activity of terminally exhausted CD8^+^ tumor-infiltrating lymphocytes (112). Recent research has confirmed that modification of CAR-T-cells to release IL-10 preserves the intact mitochondrial structure and function of T-cells, boosting oxidative phosphorylation via reliance on mitochondrial pyruvate carriers. Therefore, the growth and effectiveness of CAR-T-cells can be enhanced (113).

#### 3.5.2 Other cell-based therapies

Due to TCR-T cells have the ability to target a broader range of tumor-associated antigens, they have been developed to target intracellular tumor antigens presented by MHC molecules. Preclinical models have demonstrated that they can effectively infiltrate the TME and exert potent antitumor effects (114). The high-density ECM and abnormal vasculature in TME often impede T cell infiltration. To overcome this barrier, researchers have developed TCR-T cells expressing specific chemokine receptors, such as CXCR2 and CXCR4, to enhance their infiltration (115). Tumor cells within TME frequently evade immune detection through mechanisms such as antigen loss or downregulation of MHC expression. Multispecific TCR-T cells, including bispecific TCR-T cells, are engineered to recognize multiple tumor antigens, thereby reducing the risk of treatment failure due to antigen loss (116). In recent years, owing to the broad-spectrum antitumor activity of NK cells and their lower likelihood of developing immune tolerance, CAR-NK cell therapy has emerged as a novel immunotherapy, combining CAR technology with NK cells. CAR-NK cells avoid MHC restrictions and have minimal CRS risk. Additionally, because CAR-NK cells lack TCR expression and possess distinctive immunomodulatory characteristics, they do not induce graft-versus-host disease (GVHD) (117). Modified CAR-NK cells can better circumvent immune checkpoint mechanisms, thereby reducing the risk of tumor immune evasion (118). These features position CAR-NK cells as a next-generation cell therapy that is currently considered superior to CAR-T cells. Given that TGF-β secretion suppresses the effector functions of NK cells and reduces their recruitment, most NK cells within TME are immature and exhibit low cytotoxic capacity (119). A study in HCC demonstrated that NK cells expressing DNR II combined with CARs targeting GPC3 or AFP could effectively resist TGF-β-mediated inhibition and enhance the tumor-killing capability of CAR-NK cells (120). In a recent phase 1/2 trial for CD19-positive B cell malignancies, a CAR-NK cell expressing an anti-CD19 CAR and IL-15 (CAR19/IL-15) was designed. As anticipated, no significant CRS, neurotoxicity, or GVHD was observed. Moreover, the one-year overall survival rate reached 68% (121). Tumor cells evade immune surveillance through various mechanisms; however, TILs with their tumor-specific cytotoxic activity, can effectively recognize and kill tumor cells, particularly those that have escaped other immunotherapies. Given the functional exhaustion and limited proliferative capacity of T cells within the TME of solid tumors, Chen et al. developed TILs engineered to express CD28 and PD-1 molecules, which have shown long-term and stable efficacy in patients with renal and colorectal cancers (122). In conclusion, given the complexity of the TME, cell-based immunotherapy represents a promising opportunity in cancer immunotherapy.

### 3.6 ICIs

Cancer cells can hijack the immune system to maintain self-tolerance through inhibitory pathways that typically act as checkpoints to prevent cell death induced by T-cell activation, reduce T-cell cytotoxic effects, and minimize excessive inflammation that can damage healthy tissues (123). However, immune evasion is facilitated by immune checkpoints. Multiple immune checkpoint targets have been discovered; however, only ICIs targeting CTLA-4 and PD-1/PD-L1 have been approved and are widely available.

**Figure 3:**
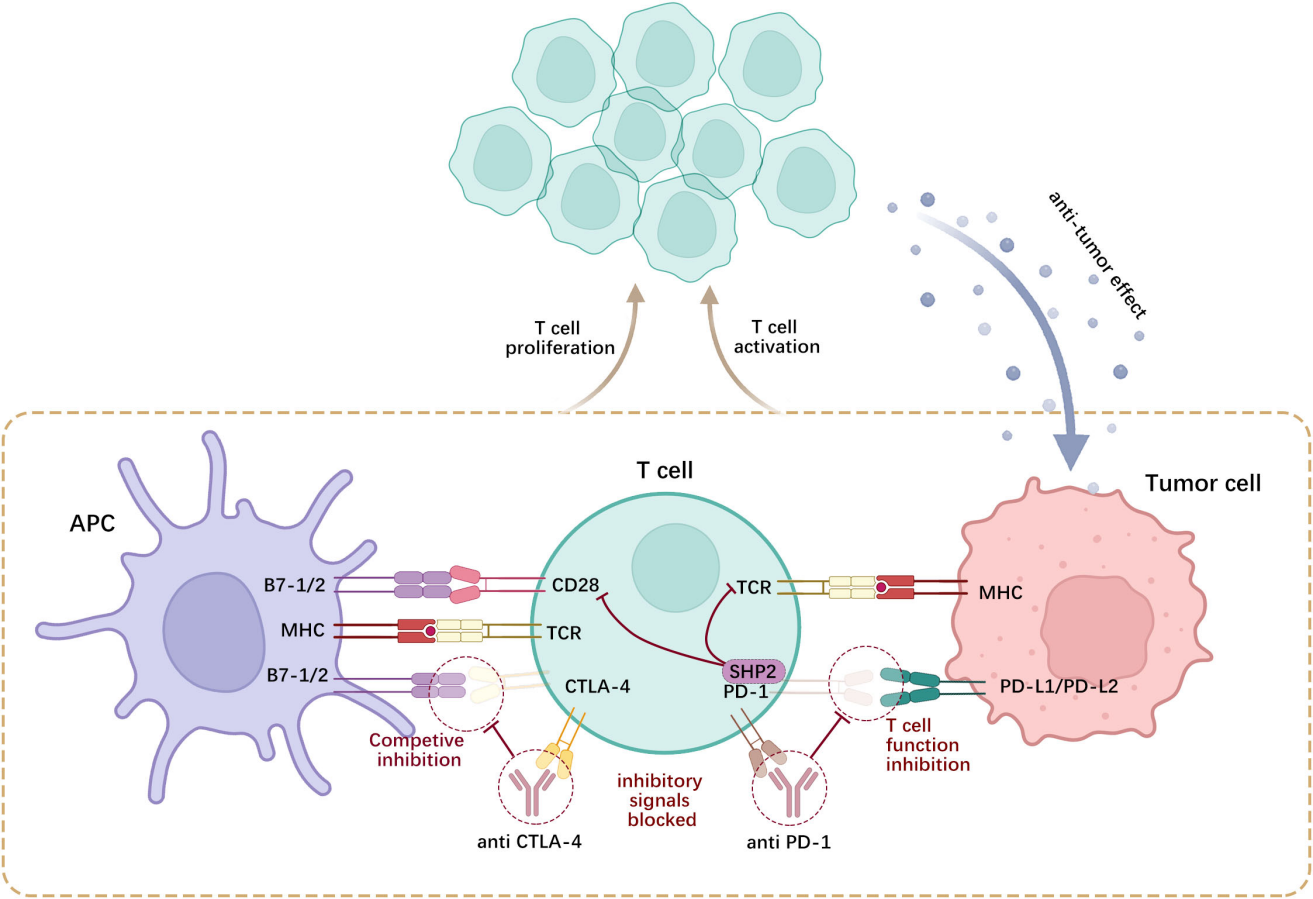
CTLA-4 and PD-1 are expressed on activated T-cells, wherein CTLA-4 competitively antagonizes CD28 and binds to B7-1/2 on the surface of APCs, while PD-1 engages with PD-L1/2 on tumor cells or APCs. Such interactions curtail the proliferation of self-reactive T-lymphocytes and the generation of cytokines.

CTLA-4 is a negative-regulatory molecule that inhibits activation signals by competitively binding to members of the B7 family of molecules (such as CD80 and CD86) (124). This competitive binding inhibits cell proliferation and promotes cell cycle arrest. In addition to its intracellular functions that weaken T-cell activity, CTLA-4 modulates T-cell activation through several extracellular mechanisms. CTLA-4, which is found in Tregs, reduces the levels of CD80/CD86 co-stimulatory molecules in antigen-presenting cells (APCs). This reduction at the molecular level impairs APCs’ capacity to effectively activate T-lymphocytes (125). PD-L1, a PD-1 ligand, plays a crucial regulatory role in the immune system by binding to PD-1. Recent evidence suggests that PD-L1 activation of PD-1 preferentially dephosphorylates the costimulatory receptor CD28 rather than the T-cell receptor (TCR) by recruiting Src homology-2-containing protein tyrosine phosphatase 2 (SHP2), thereby inhibiting T-cell function by deactivating CD28 signaling (126). Suppression of T-cell activation and proliferation results in the impaired capability of T-lymphocytes to effectively target and attack tumor cells. Moreover, PD-L1 expression leads to immune evasion by hindering the function of immune cells (127) (**Figure 3**).

Following the FDA’s endorsement of ipilimumab (anti-CTLA4) in 2011 for metastatic melanoma therapy, regulatory agencies worldwide have approved at least 11 different versions of this drug for > 90 additional indications. The first PD-1 immune checkpoint inhibitor, nivolumab, was approved in 2014, demarking a significant entry into ICI therapy. To date, the Food and Drug Administration (FDA) has approved a total of 10 monoclonal antibodies against PD-1/PD-L1. In comparison, there are currently only two approved CTLA-4 monoclonal antibodies: ipilimumab and tremelimumab. Numerous studies have demonstrated the efficacy of ICIs in treating a diverse array of cancers, including advanced melanoma (128) and non-small cell lung cancer (129).

Recent data indicate that the FDA-approved therapeutic agent tislelizumab has been used to treat adult individuals diagnosed with unresectable or metastatic esophageal squamous cell carcinoma (ESCC) who have undergone prior systemic chemotherapy. Data from a global phase 3 RATIONALE 302 trial showed that within the intention-to-treat (ITT) population, the median overall survival (OS) for patients treated with Tislelizumab was 8.6 months. In contrast, for patients receiving chemotherapy, the median OS was 6.3 months, demonstrating an extension in survival for individuals who had undergone prior systemic therapy (130). Current treatments involving ICIs face challenges, the major one being the influence of the TME. The TME hosts numerous immune inhibitory cells that can suppress T-lymphocyte function via diverse mechanisms, thereby affecting the effectiveness of ICIs (131). Therefore, researchers are developing new strategies to enhance the effectiveness of ICIs in treating cancer, including combination therapy with other anti-cancer agents such as immunotherapy, targeted therapy, and chemotherapy. Moreover, there has been an investigation into amalgamating ICIs with immune regulators to significantly enhance therapeutic results.

In a phase 3 clinical trial of patients with advanced renal cell carcinoma who had not received prior treatment, the co-administration of cabozantinib with nivolumab and ipilimumab led to a notably extended progression-free survival duration when compared with the use of nivolumab and ipilimumab as standalone treatments. The study’s experimental cohort demonstrated a 12-month progression-free survival (PFS) rate of 0.57, marginally surpassing the control group’s 0.49 rate (132). In a phase 3 study evaluating atezolizumab combined with cabozantinib versus cabozantinib alone in individuals with renal cell carcinoma who had received prior ICI therapy, researchers discovered that the median PFS did not significantly differ between the two cohorts. Joint management fails to improve clinical results and is associated with increased toxicity (133). These divergent results highlight that ICI and targeted therapy combination treatments are not necessarily suitable for all cancer patients and need to be personalized based on specific patient conditions and cancer characteristics. However, combination therapy with chemotherapy has demonstrated excellent OS rates. In a trial targeting terminal biliary tract cancer, pembrolizumab in combination with gemcitabine and cisplatin demonstrated a significant enhancement in OS compared with pembrolizumab alone. This improvement was statistically significant and clinically meaningful, suggesting that the addition of pembrolizumab can lead to superior patient outcomes. No new safety concerns have been identified regarding the use of pembrolizumab in co-administered therapy (134). Another evaluation of gastric/esophageal adenocarcinoma patients showed that the 12-month OS with ICIs combined with chemotherapy is superior to that with chemotherapy alone, supporting the co-administration of nivolumab alongside chemotherapy as a primary therapeutic option for terminal gastric or esophageal adenocarcinoma (135). Five-year follow-up results for the combination of pembrolizumab with two chemotherapeutic agents in nonsquamous cell lung cancer confirm this finding (136). Combination therapy targeting both the CTLA-4 and PD-1 pathways is more effective than monotherapy because these pathways operate via distinct mechanisms. Cadonilimab, a bispecific antibody targeting PD-1 and CTLA-4, has been tested in phase 1b/2 clinical trials in individuals with terminal cancers (137). Numerous studies have evaluated the efficacy and safety of ICIs.

### 3.7 Gut microbiota

ICIs have been used in clinical practice for a few decades and provide high survival rates for most patients with cancer. Nevertheless, this treatment does not benefit all patients because a substantial number exhibit either primary or acquired drug resistance (138). Emerging findings suggest that the gut microbiome is essential for immune therapy, as evidenced by clinical studies showing how the gut microbiota influences responses to such treatments (139). Research has indicated that the composition of the gut microbiota can influence the responses of patients with cancer to ICIs. Specific types of gut microbiota have been observed to boost the effectiveness of ICIs, thereby improving the survival rates of patients with cancer (140). Various mechanisms enable the gut microbiota to influence the effectiveness of immunotherapy, including the generation of metabolites, control of the development and function of lymphocytes, and adjustment of overall inflammatory responses.

**Figure 4:**
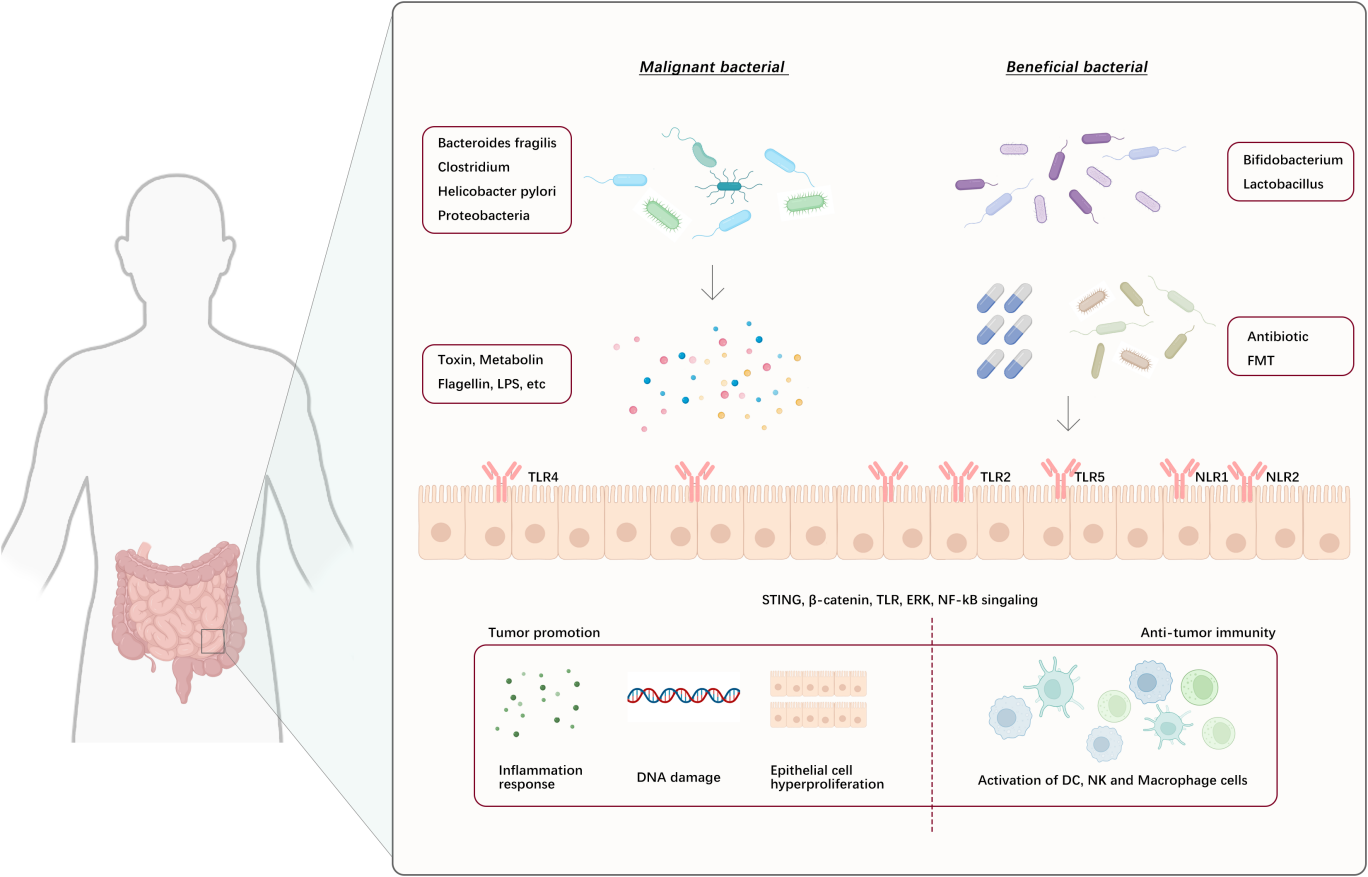
Gut microbes and their metabolites act on intestinal epithelial cells or bind to their surface pattern recognition receptors, eliciting downstream signaling cascade reflexes that lead to tumor-promoting and anti-tumor immune responses. (NLR1/2: NOD-like receptor 1 and 2, FMT: Fecal microbiota transplant).

Toll-like receptors (TLRs), a type of pattern recognition receptor, are broadly expressed in both intestinal epithelial cells (IECs) and resident immune cells in the lamina propria. Signal molecules provided by the gut microbiota, including LPS, flagellin, and bacterial peptidoglycan, interact with pattern recognition receptors to recognize and initiate downstream signaling cascades, ultimately influencing tumor immune responses (141) (**Figure 4**). Research conducted employing a mouse model of colon cancer indicated that metastasis-related protease cathepsin K (CTSK), which is secreted by tumors, can be activated by signals from the gut microbiota. This activation allows CTSK to interact with TLR4 and trigger the polarization of TAMs to the M2 phenotype through an mTOR-dependent pathway. This leads to the infiltration and metastasis of colorectal cancer (CRC) cells by activating the NF-κB pathway (142). Similarly, in an esophageal cancer study, it was identified that LPS exacerbates the invasive and migratory behaviors of esophageal cancer cells through the TLR4/NF-κB axis (143). Certain microbiota-producing toxins, such as enterotoxigenic *Bacteroides fragilis* (ETBF), promote the formation of colonic epithelial cell tumors through their toxins and IL-17 production, leading to MDSC differentiation, which upregulates arginase 1 (ARG1) and inducible nitric oxide synthase 2 (NOS2), produces NO, and inhibits T-lymphocyte proliferation, thus promoting tumorigenesis. In contrast, the gut microbiota can stimulate local or systemic immune cells to produce antitumor effects. For example, *Bifidobacterium* stimulates tumor-specific T-lymphocytes, promotes the accumulation of CD8^+^ T-lymphocytes in melanoma, and increases IFN production (144). When *Helicobacter hepaticus* (Hhep) was introduced into a CRC mouse model, there was a noticeable increase in infiltration by CTLs, resulting in suppression of tumor growth. Colonization by Hhep prompts the development of Hhep-specific T follicular helper (Tfh) cells, leading to an increase in the number of colonic Tfh cells. Additionally, the presence of Hhep supports the maturation of lymphoid structures responsible for draining tumors (145).

Regulation of the gut microbiota to improve responses to immunotherapy can be achieved through various means, including the use of probiotics or microbial consortia to enhance beneficial bacteria, the use of antibiotics to reduce levels of harmful bacteria, and fecal microbiota transplantation to introduce beneficial microbiota (146–148) (**Figure 4**). These methods have shown promise for regulating the gut microbiome in both clinical and preclinical models. Moreover, using different gut microbiota characteristics to distinguish healthy individuals from patients with cancer can potentially serve as a method for predicting how patients with cancer will respond to ICI therapy (149). While still in their initial phases, these techniques provide great enthusiasm for optimizing immunotherapeutic outcomes.

## 4 Summary and prospects

The TME is an extremely complex system involving interactions between various cell types, cytokines, and signaling pathways. Within the realm of immunotherapy, the immunoregulatory role of the TME is particularly prominent and includes immune cell activity, activation of immune escape signaling pathways, and the influence of tumor stromal cells on immune cells. Due to the heterogeneity and complexity of the TME, the effectiveness of immunotherapy remains challenging. Hence, it is crucial to thoroughly explore the interplay between immunosuppressive TME and immunotherapy to improve therapeutic effectiveness. Emphasizing that in immunotherapy, regulation and intervention involving the TME are particularly important. Optimization of suppressive elements in the immune system and boosting factors that combat tumors is essential to enhance the effectiveness of immunocytes, improve their ability to identify cancer cells, and accelerate their removal.

Future research directions should include an in-depth analysis of the interplay between immunocytes, stromal cells, and cancer cells to improve the efficacy of cancer therapies, reduce drug toxicity, and explore novel immunotherapeutic strategies such as nanotechnology, gene editing, cell therapy, and manipulation of the gut microbiota. The emerging field of genomics has highlighted the significance of the gut microbiota in immune regulation and responses to immunotherapy. Harnessing the gut microbiota-cancer-immunity axis holds promise for opening new avenues in immunotherapy, the identification of novel immune-related markers, and the development of innovative treatment approaches. Advances in technologies such as single-cell sequencing and high-throughput screening have led to the discovery of new immune-related markers and potential therapeutic targets.
